# Plasma metabolite profile for primary open-angle glaucoma in three US cohorts and the UK Biobank

**DOI:** 10.1038/s41467-023-38466-w

**Published:** 2023-05-19

**Authors:** Oana A. Zeleznik, Jae H. Kang, Jessica Lasky-Su, A. Heather Eliassen, Lisa Frueh, Clary B. Clish, Bernard A. Rosner, Tobias Elze, Pirro Hysi, Anthony Khawaja, Janey L. Wiggs, Louis R. Pasquale, UK Biobank Eye and Vision Consortium

**Affiliations:** 1 Channing Division of Network Medicine, Department of Medicine, Brigham and Women’s Hospital, Boston, MA USA; 2 Department of Medicine, Harvard Medical School, Boston, MA USA; 3 Department of Nutrition, Harvard T.H. Chan School of Public Health, Boston, MA USA; 4 Department of Epidemiology, Harvard T.H. Chan School of Public Health, Boston, MA USA; 5 Broad Institute of Massachusetts Institute of Technology and Harvard, Cambridge, MA USA; 6 Department of Biostatistics, Harvard T.H. Chan School of Public Health, Boston, MA USA; 7 Department of Ophthalmology, Schepens Research Eye Institute of Massachusetts Eye and Ear, Boston, MA USA; 8 Department of Ophthalmology, King’s College London, London, UK; 9 Department of Twin Research & Genetic Epidemiology, King’s College London, London, UK; 10 St. Thomas’ Hospital, London, UK; 11 National Institute for Health and Care Research Biomedical Research Centre, Moorfields Eye Hospital, London, UK; 12 National Institute for Health and Care Research Biomedical Research Centre, Institute of Ophthalmology, University College London, London, UK; 13 Department of Ophthalmology, Massachusetts Eye and Ear, Boston, MA USA; 14 Department of Ophthalmology, Icahn School of Medicine at Mount Sinai, New York, NY USA

**Keywords:** Optic nerve diseases, Predictive markers

## Abstract

Glaucoma is a progressive optic neuropathy and a leading cause of irreversible blindness worldwide. Primary open-angle glaucoma is the most common form, and yet the etiology of this multifactorial disease is poorly understood. We aimed to identify plasma metabolites associated with the risk of developing POAG in a case-control study (599 cases and 599 matched controls) nested within the Nurses’ Health Studies, and Health Professionals’ Follow-Up Study. Plasma metabolites were measured with LC-MS/MS at the Broad Institute (Cambridge, MA, USA); 369 metabolites from 18 metabolite classes passed quality control analyses. For comparison, in a cross-sectional study in the UK Biobank, 168 metabolites were measured in plasma samples from 2,238 prevalent glaucoma cases and 44,723 controls using NMR spectroscopy (Nightingale, Finland; version 2020). Here we show higher levels of diglycerides and triglycerides are adversely associated with glaucoma in all four cohorts, suggesting that they play an important role in glaucoma pathogenesis.

## Abstract (web summary)

Primary open-angle glaucoma is a leading cause of blindness. Here, the authors report higher plasma levels of diglycerides and triglycerides in samples collected prior to diagnosis, particularly in cases presenting with vision loss near fixation.

## Introduction

Glaucoma is a progressive optic neuropathy that is a leading cause of irreversible blindness worldwide^1^. Primary open-angle glaucoma (POAG) is the most common form, and yet the etiology of this multifactorial disease is poorly understood. Genome-wide association studies have identified >120 genetic loci for POAG^2^, and the finding of multiple genes in various pathways suggests that there is a complex metabolic network that affects optic nerve health.

The metabolome is the set of small molecule metabolites that are critical for the growth and maintenance of cells and tissues^3,4^, and represent the end-products of environmental factors and gene expression associated with responses to such factors. Metabolomics platforms quantify blood metabolites and can be used to evaluate the etiologic role of metabolic alterations. A recent systematic review^5^ identified several studies which found novel markers for prevalent POAG/glaucoma by evaluating metabolomic profiles measured in either serum^6–8^, plasma^9–11^, aqueous humor^12–15^, tear^16^, and optic nerve^17^ samples. However, these studies are limited by relatively small sample sizes (the largest study included 211 cases and 295 controls^7^), focus on treated POAG cases^9–11,13–18^ which may result in bias due to treatment, and use of convenience controls with other eye conditions^13–15,19,20^. Additionally, the use of prevalent cases may be problematic for the discovery of changes related to early disease, as consequences of advanced disease or treatment are likely to impact circulating metabolite profiles in glaucoma. Our study included 599 incident cases and 599 matched controls in a nested case-control study of pre-diagnostic circulating plasma metabolites from ~10 years before POAG diagnosis, and to confirm the findings, we evaluated the metabolomic data in prevalent glaucoma cases from the UK Biobank.

Here, we show that higher levels of diglycerides and triglycerides are adversely associated with incident POAG in three health professional cohorts with stronger associations for POAG with paracentral visual field (VF) loss. We confirmed the adverse associations for glycerides in a cross-sectional analysis performed in the UK Biobank.

## Results

### Study population—Nurses’ Health Study (NHS), NHSII, and Health Professional Follow-up Study (HPFS)

Among 599 cases, 74.3% were female, with a mean age at blood draw of 58.0 (standard deviation (SD) = 8.0) years and at diagnosis of 68.3 (SD = 9.2) years. The mean time between blood draw to diagnosis was 10.3 years. Controls were similar to cases for the matching factors. Distributions of POAG risk factors were generally in the expected directions for cases and controls (Table 1).

**Table 1:** Participant characteristics in the Nurses’ Health Study, Nurses’ Health Study II, and Health Professionals’ Follow-up Study at the time of blood collection^a^

	Cases	Controls
	*n* = 599	*n* = 599
Age, years^b^ (SD)	58.0 (8.0)	57.9 (7.9)
Men, *n* (%)^b^	154 (25.7)	154 (25.7)
Race/ethnicity, *n* (%)^b^		
African-American	8 (1.3)	3 (0.5)
Asian	6 (1.0)	1 (0.2)
Hispanic	8 (1.3)	4 (0.7)
Non-Hispanic White	577 (96.3)	591 (98.7)
Time of blood draw, *n* (%)^b^		
12:00 a.m.–7:59 a.m.	70 (12.2)	74 (12.7)
8:00 a.m.–9:59 a.m.	293 (51.2)	321 (55.2)
10:00 a.m.–11:59 a.m.	124 (21.7)	121 (20.8)
12:00 p.m.–11:59 p.m.	85 (14.9)	65 (11.2)
Season of blood draw, *n* (%)^b^		
Winter	137 (22.9)	126 (21.0)
Spring	223 (37.2)	206 (34.4)
Summer	202 (33.7)	218 (36.4)
Autumn	37 (6.2)	49 (8.2)
Smoking status, *n* (%)		
Never	305 (51.3)	301 (50.8)
Past	244 (41.0)	248 (41.8)
Current	46 (7.7)	44 (7.4)
Body mass index, kg/m^2^ (SD)	25.4 (4.7)	25.5 (4.2)
Physical activity, MET-h/week (SD)	23.2 (34.5)	22.4 (29.9)
Family history of glaucoma, *n* (%)	180 (31.1)	81 (14.4)
Age at menopause, years (SD)	49.5 (3.6)	49.4 (3.4)
Socio-economic status index score (SD)	0.0 (4.9)	0.0 (4.8)
Nitrate intake, mg/day (SD)	152.3 (88.0)	157.7 (83.1)
Alcohol intake, g/day (SD)	7.1 (11.2)	6.9 (12.0)
Caffeine intake, mg/day (SD)	263.1 (233.6)	261.8 (232.5)
Alternative Healthy Eating Index score (SD)^c^	48.3 (11.0)	48.7 (11.0)
Total caloric intake, kcal/day (SD)	1841.0 (539.5)	1832.7 (550.0)
Hypertension, *n* (%)	153 (25.5)	141 (23.5)
High cholesterol, *n* (%)	193 (32.2)	188 (31.4)
Diabetes, *n* (%)	24 (4.0)	19 (3.2)

*MET* metabolic equivalent of task.

^a^Values are means ± standard deviation (SD) or numbers (percentages) and are based on those with non-missing values.

^b^Cases and controls were 1:1 matched based on age (all >40 years), cohort/gender, month and year of blood collection, time of day of blood draw, fasting status (> or ≤8 h), race/ethnicity, and among women: additional matching on menopausal status and hormone therapy use at blood draw (premenopausal, postmenopausal and on hormone therapy (HT), postmenopausal and not on HT, missing/unknown) and at glaucoma diagnosis. All cases and controls reported eye exams in the index period of the matched cases’ diagnosis date.

^c^Alternative Healthy Eating Index scores (excluding alcohol) range from 0 to 100; a higher score reflects a healthier diet.

### Relation between individual metabolites and POAG in NHS, NHSII, and HPFS

**Fig. 1:**
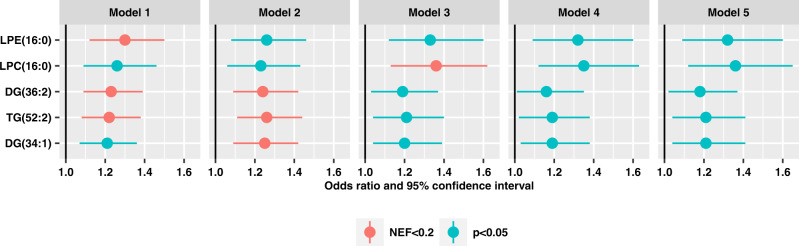
Individual metabolites among the *n* = 369 metabolites evaluated that were significant across the various nested multiple conditional logistic regression models of primary open-angle glaucoma (599 cases and 599 controls in NHS/NHSII/HPFS). Data are presented as odds ratios and 95% confidence intervals estimated with conditional logistic regression models. **Model 1**: basic model, adjusting for matching factors only (see Table 1); **Model 2** (factors that affect metabolite levels and matching factors as matching was imperfect): Model 1 plus age, gender, smoking status, BMI, physical activity, time of day of blood draw, month of blood draw, fasting status; **Model 3** (established risk factors for primary open-angle glaucoma (POAG)): Model 2 plus family history of glaucoma, socioeconomic index based on census tract data, race/ethnicity and age at menopause; **Model 4** (potential modifiable dietary risk factors for POAG): Model 3 plus nitrate intake, caffeine intake, alcohol intake, caloric intake; **Model 5** (systemic comorbidities/drugs suggested to be associated with POAG in some studies): Model 4 plus hypertension, high cholesterol, diabetes, and oral steroid use. LPC (16:0) and LPE (16:0) were assessed among women only. LPE lysophosphatidylethanolamine; LPC lysophosphatidylcholine; DG diglyceride; TG triglycerides. Exact species can be found using the HMDB identification number listed in the supplement. All statistical tests are two-sided, and we accounted for multiple comparisons by using *p* values based on the number of effective (NEF) tests. Source data with exact values are provided as a Source Data file.

Supplementary Data 1 present the individual associations between the 369 metabolites and POAG. Five metabolites that were significant at number of effective tests corrected *p* (NEF) < 0.2 in any of the models (Model 1 through Model 5) are depicted in Fig. 1. In Model 1 (model incorporating matching), two diglycerides (DG(36:2) and DG(34:1)), one triglyceride (TG(52:2)), one lysophosphatidylcholine (LPC(16:0)), and one lysophosphatidylethanolamine (LPE(16:0)) were adversely associated with POAG; of these, LPE(16:0), DG(36:2), and TG(52:2) were significant with NEF < 0.2. In successive models, the associations were generally similar, although the significance was attenuated with additional adjustment for covariates. In Model 2, where we more finely adjusted for matching factors (e.g., fasting status, time of day) and other major determinants of variability in metabolites, such as BMI, only TG(52:2), DG(36:2) and DG(34:1) exhibited adverse associations with NEF < 0.2. However, in successive models of Model 3 and 4 (addition of POAG established and suspected risk factors), and Model 5 (further adjust for co-morbidities), we observed that while no metabolite exhibited significance at the NEF < 0.2 level (except for LPC(16:0) in Model 3), all were nominally significant and the direction of associations for the metabolites was similar (*p* < 0.05). None of the metabolites associated with POAG showed statistically significant (NEF < 0.2) heterogeneity by gender in any of the models, thus all subsequent main results are shown for men and women together. However, LPC (16:0) and LPE (16:0) failed our delayed blood processing pilot study among men, thus the results for these two metabolites shown in Fig. 1 represent associations among women only.

### Relation between metabolite classes and POAG in NHS, NHSII, and HPFS

**Fig. 2:**
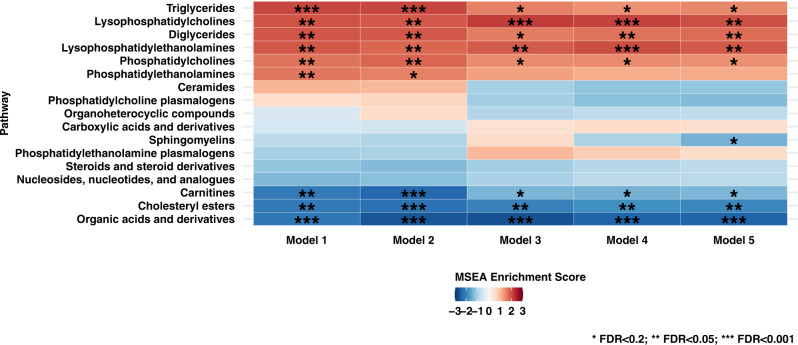
Metabolite classes (*n* = 17) evaluated in various nested multiple conditional logistic regression models of primary open-angle glaucoma (POAG; 599 cases and 599 controls in NHS/NHSII/HPFS). Metabolite Set Enrichment Analysis was used to estimate enrichment scores based on estimates from conditional logistic regression models. **Model 1**: basic model, adjusting for matching factors only (see Table 1); **Model 2** (factors that affect metabolite levels and matching factors as matching was imperfect): Model 1 plus age, gender, smoking status, BMI, physical activity, time of day of blood draw, month of blood draw, fasting status; **Model 3** (established risk factors for primary open-angle glaucoma (POAG)): Model 2 plus family history of glaucoma, socioeconomic index based on census tract data, race/ethnicity, and age at menopause; **Model 4** (potential modifiable dietary risk factors for POAG): Model 3 plus nitrate intake, caffeine intake, alcohol intake, caloric intake; **Model 5** (systemic comorbidities/drugs suggested to be associated with POAG in some studies): Model 4 plus hypertension, high cholesterol, diabetes, and oral steroid use. * False Discovery Rate (FDR) < 0.2; ** FDR < 0.05; *** FDR < 0.001. Alkaloids and derivatives could not be evaluated because not all quinine models, one of the three metabolites belonging to this class, converged in the individual metabolite analyses. All statistical tests are two-sided, and we accounted for multiple comparisons by using FDR. Source data with exact values are provided as a Source Data file.

Overall, the relation between metabolite classes and POAG yielded more robust results. Figure 2 shows the results of 17 metabolite classes in relation to POAG. Model 1 showed 9 classes that were associated: 6 lipid metabolite classes, namely TGs, LPCs, DGs, LPEs, phosphatidylcholines (PCs), and phosphatidylethanolamine (PEs) were adversely associated with POAG risk, while cholesteryl esters, carnitines, and organic acids and derivatives (which includes amino acids) were inversely associated with POAG risk (false discovery rate (FDR) < 0.05 for all). In Model 2, results were similar, although for PEs, the FDR was <0.2. In subsequent models, which include additional covariates, the adverse associations with LPCs, LPEs, and DGs and the inverse associations with cholesteryl esters and organic acids and derivatives (which includes amino acids) were robust (FDR < 0.05 for all in Model 5). For PEs, the FDR was ≥0.2. The adverse relation with POAG for PCs and TGs and the inverse relation for carnitines was still significant (FDR was <0.2). Furthermore, in Model 5, an inverse association between sphingomyelins and POAG was observed (FDR < 0.2).

### Relation between metabolite classes and POAG subtypes defined by visual field loss patterns in NHS, NHSII, and HPFS

**Fig. 3:**
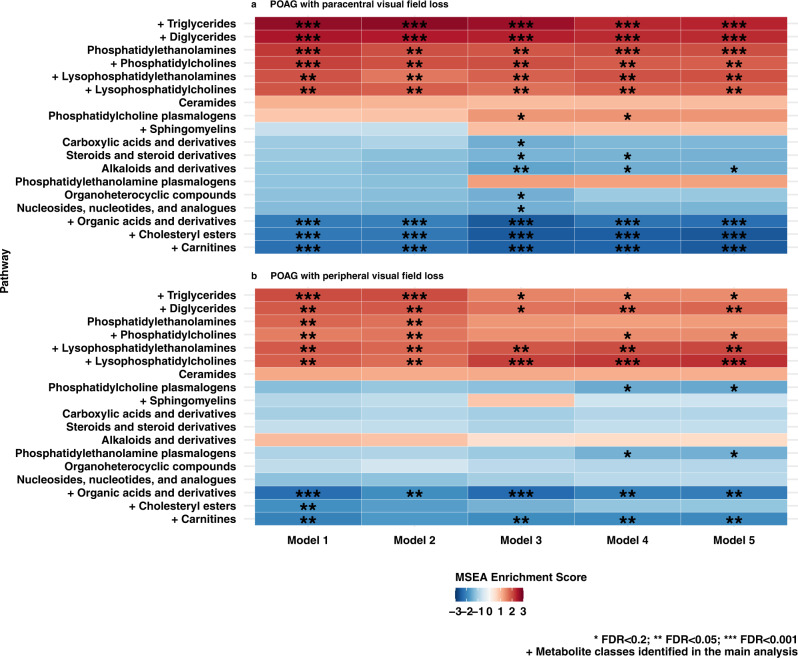
Metabolite classes (*n* = 18) evaluated in various nested multiple logistic regression models of POAG subtypes defined by visual field loss patterns in NHS/NHSII/HPFS. Results for POAG with paracentral visual field loss (178 cases and 599 controls) are shown in (**a**) while results for POAG with peripheral visual field loss (331 cases and 599 controls) are shown in (**b**). Metabolite Set Enrichment Analysis was used to estimate enrichment scores based on estimates from logistic regression models. **Model 1**: basic model, adjusting for matching factors only; **Model 2** (factors that influence metabolites): age, smoking status, BMI, physical activity, time of day (as matching imperfect), month of blood draw (season, as matching imperfect); **Model 3** (established risk factors for POAG): Model 1 plus family history of POAG, SES, race, age at menopause; **Model 4** (established risk factors for POAG): Model 3 plus nitrate intake, caffeine intake, alcohol intake, alternate healthy eating index, caloric intake; **Model 5** (co-morbidities/drugs that have been associated with POAG): Model 4 plus hypertension, high cholesterol, diabetes, oral/inhaled steroid use. All statistical tests are two-sided, and we accounted for multiple comparisons by using FDR. Source data with exact values are provided as a Source Data file. * False Discover Rate (FDR) < 0.2; ** FDR < 0.05; *** FDR < 0.001.

POAG is multifactorial and clinically heterogeneous. Investigating heterogeneity in VF loss patterns for POAG may provide new etiologic insights as different types of optic nerve damage manifest as distinct VF loss patterns. For example, glaucomatous paracentral scotomas have been associated with more systemic risk factors compared to peripheral VF loss^21–23^. Therefore, we separately evaluated the associations between metabolite classes and POAG defined by VF loss patterns (paracentral (Fig. 3a) versus peripheral VF loss (Fig. 3b)). Of the 599 cases, VF loss patterns derived from Humphrey visual field test were available in 509 cases. As shown in Fig. 3a and Fig. 3b, overall, more metabolite classes (9 classes versus 5 classes) were significantly associated with POAG with paracentral VF loss versus peripheral VF loss. In addition to the three classes associated with peripheral VF loss, namely DGs, LPCs, and LPEs, for POAG with paracentral VF loss, the classes of TGs, PEs, and PCs were also adversely associated at FDR < 0.05 (Model 5). Notably, the adverse relation between TGs/DGs/PEs and POAG with paracentral VF loss was significant at the FDR < 0.001 level. Furthermore, in addition to carnitines and organic acids and derivatives being inversely associated with POAG with peripheral VF loss, cholesteryl esters were also inversely associated with POAG with paracentral VF loss at FDR < 0.05 (Model 5).

### Secondary analyses in NHS, NHSII, and HPFS

Prior studies of TG in relation to health outcomes^24,25^ have observed differing associations by the degree of saturation and number of carbon atoms. Therefore, we further plotted the associations with incident POAG as a function of the number of double bonds and carbon atoms in TGs (Supplementary Fig. 1). Interestingly, as found with diabetes and cardiovascular disease^24,25^, in general, those TG with fewer carbon atoms and double bonds were adversely associated with POAG risk while those with higher carbon atoms and double bonds were inversely associated with POAG risk.

To evaluate whether associations differed by subgroups, in exploratory analyses, we assessed whether results varied by age (Supplementary Fig. S2), gender (Supplementary Fig. S3), BMI (Supplementary Fig. S4), time to diagnosis (Supplementary Fig. S5), and family history of glaucoma (Supplementary Fig. S6). Although no major differences were observed in various subgroups, there were some suggestions that the associations were strongest in those with BMI > 25 kg/m^2^ and those diagnosed closer in time to blood draw (within a decade).

### UK Biobank

To assess whether the associations observed in NHS/NHSII/HPFS might also be observed in the UK Biobank, we conducted metabolomic analyses of the outcome of glaucoma, defined based on self-reported glaucoma, use of glaucoma medications and ICD codes (2238 glaucoma cases and 44723 non-cases). In general, glaucoma cases were older, had more diabetes, and higher systolic blood pressure than controls (Table 2).

**Table 2:** UK Biobank participant characteristics at the time of blood draw

	Glaucoma Cases	Controls
	*n* = 2238	*n* = 44723
Age, years (SD)	61.2 (6.4)	56.8 (8.0)
Men, *n* (%)	1178 (52.6)	20369 (45.5)
Smoking status, *n* (%)		
Never	1164 (52.3)	24711 (55.5)
Past	853 (38.3)	15315 (34.4)
Current	208 (9.3)	4517 (10.1)
Physical activity, MET-h/week (SD)	2684.5 (2734.1)	2624.0 (2670.8)
Body mass index, kg/m^2^ (SD)	27.8 (4.5)	27.3 (4.5)
Race/Ethnicity, *n* (%)		
Asian	64 (2.9)	1272 (2.8)
Black	76 (3.4)	1078 (2.4)
Other	49 (2.2)	1034 (2.3)
White	2038 (91.5)	41136 (92.4)
Coffee consumption, cups/day (SD)	1.9 (1.7)	1.9 (1.8)
Tea consumption, cups/day (SD)	3.1 (2.0)	3.1 (2.1)
Alcohol intake, *n* (%)		
1–2 times a week only	539 (24.1)	11171 (25.0)
Systolic blood pressure, mm Hg (SD)	141.2 (17.9)	137.3 (18.1)
Diabetes, *n* (%)	256 (11.4)	3001 (6.7)
Coronary artery disease, *n* (%)	210 (9.4)	2342 (5.2)
Cholesterol, mmol/l (SD)	5.6 (1.2)	5.7 (1.1)

*SD* standard deviation.

Values are means ± SD (SD) or percentages and are based on those with non-missing values.

**Fig. 4:**
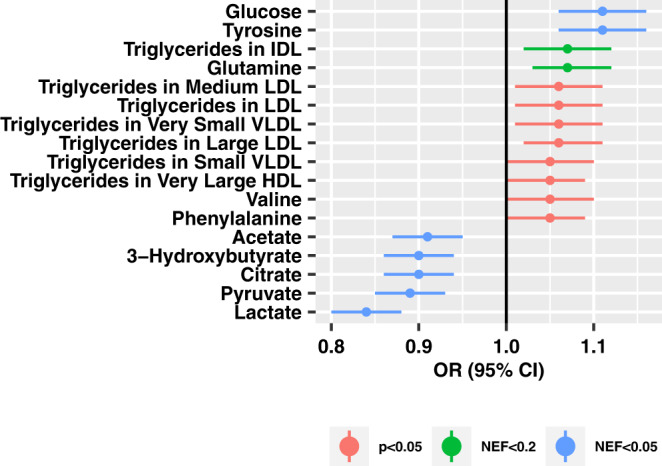
Individual metabolites (out of *N* = 168) that were at least nominally significantly associated with prevalent glaucoma in the UK Biobank (2238 cases, 44,723 controls). Data are presented as odds ratios and 95% confidence intervals estimated with logistic regression models. The logistic regression model includes adjustment for age, gender, smoking status, physical activity, BMI, ethnicity, spherical equivalent, coffee consumption, tea consumption, alcohol intake, systolic blood pressure, cholesterol level, diabetes, coronary artery disease, and statin use. All statistical tests are two-sided, and we accounted for multiple comparisons by using p-values based on number of effective tests (NEF). Source data with exact values are provided as a Source Data file.

In multivariable-adjusted analyses of individual metabolites (Fig. 4**;** Supplementary Data 2), we observed that 6 TG metabolites were nominally associated with higher glaucoma risk (*p* < 0.05). Tyrosine (NEF < 0.05), glucose (NEF < 0.05), glutamine (NEF<0.2), and one TG metabolite (NEF < 0.2) were also significantly associated with higher glaucoma risk. Specific organic acids and derivatives, such as acetate, 3-hydroxybutyrate, citrate, pyruvate, and lactate were inversely associated with glaucoma (NEF < 0.05). Notably, data on glucose, acetate, 3-hydroxybutyrate, citrate, pyruvate, and lactate were not available in NHS/NHSII/HPFS; however, there were null associations between tyrosine, valine, glutamine, and phenylalanine with POAG (Supplementary Data 1).

**Fig. 5:**
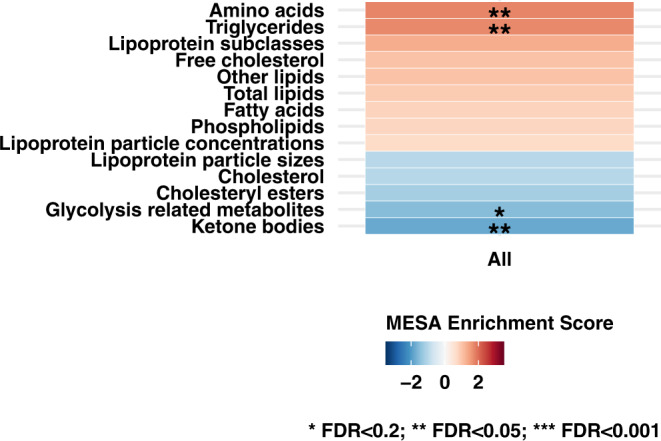
Metabolite classes (*N* = 14) evaluated with prevalent glaucoma in the UK Biobank (2238 cases, 44,723 controls). Metabolite Set Enrichment Analysis was used to estimate enrichment scores based on estimates from logistic regression models. Multiple logistic regression model includes age, gender, smoking status, physical activity, BMI, ethnicity, spherical equivalent, coffee consumption, tea consumption, alcohol intake, systolic blood pressure, cholesterol level, diabetes, coronary artery disease, and statin use. All statistical tests are two-sided, and we accounted for multiple comparisons by using FDR. Source data with exact values are provided as a Source Data file. *FDR < 0.2; **FDR < 0.05; ***FDR < 0.001.

Figure 5 shows results from evaluating metabolite classes in glaucoma in the UK Biobank. Amino acids and TGs were positively associated while ketone bodies were inversely associated with glaucoma (FDR < 0.05). Glycolysis-related metabolites were inversely associated with glaucoma at FDR < 0.2.

## Discussion

Pre-clinical plasma metabolite profiling indicates that higher levels of DGs and TGs are adversely associated with incident POAG in 3 health professional cohorts with stronger associations for POAG with paracentral VF loss. The adverse associations for glycerides were confirmed in a cross-sectional analysis performed in the UK Biobank. While this study was the first to evaluate the relation between pre-diagnostic plasma metabolites, the replication of our findings in a prevalent glaucoma dataset supports a role for altered lipid regulation in glaucoma.

A systematic review^5^ identified 13 studies to date on the metabolomics of open-angle glaucoma. Of these, three evaluated serum^6,7,26^, and three evaluated plasma^9–11^ while others evaluated aqueous humor^12–15^, tear^27^, and optic nerve^17^ samples. These studies have collectively assessed ~140 different metabolites. Compared to existing studies (where the largest study included 211 cases and 295 controls^7^), our study was unique in that the sample size in NHS/NHSII/HPFS was the largest to date (599 cases and 599 controls), did not use a convenience control sample (e.g., those with cataract or other non-glaucoma eye conditions) and importantly, evaluated pre-diagnostic plasma collected a mean of 10.3 years before POAG diagnosis which is unaffected by manifest disease or treatment. In addition, our study evaluated metabolomic and glaucoma data from the UK Biobank, to provide independent confirmation of findings, although the metabolomics platforms did not overlap substantially. Nonetheless, the nominal replication of adverse associations between selected lipid species with glaucoma is remarkable as the assay systems were fundamentally different: LC-MS/MS in NHS/NHSII/HPFS vs NMR spectroscopy in UK Biobank. Of note, the systematic review identified 12 metabolites associated with open-angle glaucoma in at least two studies; of these, two were phospholipids (PCs), two were organic acids and derivatives (including amino acids), and two were carnitines^5^. Our results are consistent with prior studies in identifying phospholipids and various organic acids and derivatives in the NHS/NHSII/HPFS and UK Biobank, while also showing support for the role of lipids, particularly diglycerides (DGs), triglycerides (TGs), and other lipids (e.g., cholesteryl esters).

### Triglycerides and cholesteryl esters

The relation between TGs and glaucoma has been conflicting, with studies showing an adverse association^28^, inverse association^29^, and null association (Mendelian randomization study^30^). A systematic review^31^ of 12 studies reported a significantly higher risk of open-angle glaucoma with hyperlipidemia as well as hypertriglyceridemia (OR = 1.42; 95%CI 1.04, 1.93; based on pooling of 4 studies). Our study confirmed this adverse association between higher TGs and POAG risk in two populations. This finding is consistent with the discovery of POAG loci near genes related to cholesterol metabolism (*ABCA1* and *CAV1/2)*. One biological mechanism for this association may be IOP-related: hypertriglyceridemia can lead to increased blood viscosity, which causes elevation of episcleral venous pressure^32,33^ and higher IOP. Indeed, a review^31^ reported that higher TG level was significantly associated with modest increases in IOP while another study suggests POAG patients have lower optic nerve blood flow which may be related to higher blood viscosity in these patients^34^. Furthermore, Shoji et al. found reduced nail fold capillary blood velocity that correlated with altered ophthalmic hemodynamics in POAG patients^35^. Interestingly, we observed that the relationship with TGs was complex. TGs that are shorter and more saturated were adversely associated while those that are longer and are more unsaturated were inversely associated with POAG, possibly because such TGs may be markers of a healthier cardiometabolic profile^25^. Finally, TGs showed stronger associations with POAG with paracentral VF loss than that with peripheral loss, further underscoring the observation that this subtype is more strongly associated with systemic factors. As for cholesteryl esters, there is limited data in relation to glaucoma; however, it is known that the ratio of cholesterol to esterified cholesterol is altered with age and in several neurodegenerative diseases^36^.

### Phospholipids and sphingomyelins

We observed adverse associations between several phospholipid classes and POAG risk in NHS, NHSII, and HPFS. The phospholipid metabolite class had a positive, but non-significant enrichment score in UK Biobank. PCs/PEs and their partial hydrolysis products, LPCs/LPEs, are implicated in aging^37–39^, as well as type 2 diabetes^40^, and play key roles in mitochondrial dynamics^41^. PEs and PCs are abundant in membrane structures that facilitate vacuolar delivery and cytokinesis. However, LPCs are pro-inflammatory and pro-oxidant^42^, and they may play a role in the autotaxin lysophosphatidic acid pathway in IOP regulation^14,15,43–46^. Our results are consistent with other targeted lipidomic studies of glaucoma that have revealed higher specific phospholipids in blood^5^, the trabecular meshwork, or aqueous humor of glaucomatous eyes^44,45,47,48^. Sphingomyelins, a type of sphingophospholipid, were inversely associated with POAG risk in NHS/NHSII/HPFS (Fig. 2) but not with prevalent glaucoma in the UK Biobank. Sphingomyelins (SMs) are abundant in the membranes of nerve cells and brain cells. SM metabolism was significantly altered in the plasma of glaucoma cases^5^ and shows complex tissue-specific changes: Lower total SM amount, corresponding to higher SM percentage of total lipids, was reported in the trabecular meshwork of glaucomatous eyes^49^ while SM levels were higher in the aqueous humor of glaucomatous eyes^50^. No changes in SM levels were observed in the optic nerve of glaucoma cases^51^.

### Mitochondrial dysfunction, carnitines, and organic acids and derivatives

Mitochondrial dysfunction has been hypothesized as a key component of POAG pathophysiology^52–58^. Plasma metabolomic markers of mitochondrial function may include acylcarnitines, which transport acyl-groups (organic acids and fatty acids) into the mitochondria so that they can be broken down to produce energy in a process known as beta-oxidation. In our study, POAG cases had lower levels of acylcarnitines, particularly the long-chain acylcarnitines. Interestingly, high-fructose diets were associated with a decrease in long-chain acylcarnitines, suggesting a decrease in mitochondrial β-oxidation and an increase in lipid peroxidation, which also led to higher TGs^59^. Carnitine has also been shown to be neuroprotective in a glaucoma animal model^60^. This is consistent with several cross-sectional studies of glaucoma that have also identified alterations in the carnitine pool^10,14,27^.

Regarding mitochondrial health, greater use of ketone bodies as an energy source, such as higher 3-hydroxybutyrate versus glucose may be important for optic nerve head homeostasis. In the UK Biobank, higher levels of ketone bodies and specifically 3-hydroxybutyrate were inversely associated with POAG risk. Ketone bodies have been associated with greater mitochondrial efficiency and have neuroprotective properties^61^. This is consistent with a large genetic pathway analysis study that identified the butanoate pathway, which is involved in generating precursors for ketone bodies, as being important for POAG etiology^62^, a study that observed suggestive lower risk of POAG with paracentral VF loss with a plant-based low carbohydrate diet^63^, and another study analyzing genes encoding mitochondrial proteins that implicated lipid and carbohydrate metabolism as being important in POAG^64^.

Among the most inversely associated organic acids and derivatives were N1-Acetylspermidine and N1,N12-Diacetylspermine (Supplementary Data 1). Spermine and spermidine are amines that also regulate mitochondrial membrane potential and have neuroprotective effects^10^. These metabolites have also been inversely associated with POAG in the study by Leruez et al.^10^. Tyrosine is another metabolite that warrants further study as it was adversely associated with glaucoma in the UK Biobank and the study by Leruez et al.^10^.

A limitation of our data is that our NHS/NHSII/HPFS and UK Biobank study population has a high percentage of White participants; therefore, our findings may not be generalizable to other populations with different races and ethnicity compositions. Additionally, there may have been residual confounding by other unmeasured factors. A further potential limitation is that our NHS/NHSII/HPFS blood samples were stored for a long time and were collected at a single timepoint. However, we follow recommended best practices: all samples are stored in the vapor phase of liquid nitrogen freezers (temperature ≤–130 °C), which are alarmed and monitored 24 h a day. Although the freezer nitrogen levels are automatically monitored, we also check them manually each week. As samples from cases and controls were collected at the same time, storage time will have similar effects on both cases and controls and result in a bias towards the null. Furthermore, findings from metabolomic studies using samples from NHS and NHSII recapitulated published findings from human and animal studies^65–67^. Additionally, we showed that most of the measured metabolites in NHS/NHSII/HPFS are reasonably stable for over 10 years;^68,69^ furthermore, we included only metabolites with good correlations for within-person stability over at least 1 year.

There are other study design issues to consider. The UK Biobank included participants with prevalent glaucoma, which would have been comprised mostly of POAG cases, but may have also included those with other glaucoma types or glaucoma suspects. Furthermore, in the UK Biobank, metabolite levels may have been influenced by glaucoma treatment. The outcome heterogeneity between US and UK participants would have biased findings towards the null and thus, may partially explain why not all findings from NHS, NHSII, and HPFS were validated in the UK Biobank. Additionally, the overlap in the metabolites in the two samples was minimal as distinct technologies were used to measure metabolomics in the NHS/NHSII/HPFS (Liquid Chromatography Mass Spectrometry [LC-MS]) and the UK Biobank (Nuclear magnetic resonance [NMR] spectroscopy). Specifically, only nine metabolites directly overlap in both datasets. In NHS/NHSII/HPFS, we assessed multiple individual triglycerides based on the length and unsaturation of fatty acid chains. In contrast, in the UK Biobank, triglycerides are quantified by the type and size of their lipoprotein carrier. However, this limitation could be a strength in that the results from studies using different technologies complemented each other, further supporting our study conclusions. However, the confirmation of the associations with TGs provided support to the prospective associations observed in NHS/NHSII/HPFS. Finally, current metabolomic profiling methods, including LC-MS and NMR, measure only a subset of the metabolome. In fact, only around 25% of all metabolites are known and have a pathway annotation. This makes the field of metabolomics exciting as our ability to annotate yet unknown markers can advance our knowledge of various endpoints, including glaucoma.

Our study had several strengths. To our knowledge, our study in NHS/NHSII/HPFS is the first study assessing the associations of pre-diagnostic metabolites and POAG risk, with a relatively large sample size of 599 cases and 599 matched controls. Additional strengths include the detailed covariate information and long time between blood draw and diagnosis date (mean=10.3 years) and the availability of the UK Biobank data to confirm findings. Furthermore, the stronger metabolite associations for POAG with paracentral VF loss among health professionals indicate that lipid dysregulation and mitochondrial function play a particularly important role in this disease subtype.

Overall, these results provide new insights into the etiology of POAG. Our data implicate dysregulation in lipid metabolism and mitochondrial function in glaucoma etiology and suggest new targets for glaucoma prevention or therapies.

## Methods

### Study population—Nurses’ Health Study (NHS), NHSII, and Health Professional’s Follow-up Study (HPFS)

We conducted a nested case-control study in the United States-based NHS, NHSII, and HPFS cohorts. The NHS was initiated in 1976 with 121,700 female registered nurses aged 30–55 years at enrollment; the NHSII began in 1989 with 116,429 female registered nurses aged 25-42 years, and the HPFS was launched in 1986 with 51,529 male health professionals aged 40–75 years. Participants completed biennial questionnaires that asked about lifestyle and medical conditions, such as glaucoma. POAG cases were identified among participants who self-reported a physician diagnosis of glaucoma on biennial questionnaires. To confirm the self-reports of glaucoma, we asked participants if we could obtain confirmatory medical information from their treating physicians. All eye care providers were requested to send all available visual fields (VFs) and were mailed a supplementary questionnaire to complete and return. A glaucoma specialist (LRP) reviewed the questionnaire (or medical records sent instead of questionnaires) as well as the VFs in a standardized manner. This questionnaire included items about untreated maximum intraocular pressure (IOP), any secondary causes of high untreated IOP, the filtration angle, structural features of the optic nerve, glaucoma surgery, any VF loss, and any secondary conditions that may cause VF loss. We required cases to have at least two reliable VFs (≤20% for false negative rate and false positive rate and ≤33% for fixation loss rate) that showed reproducible defects due to glaucoma, non-occludable angles in both eyes, and no secondary causes of IOP elevation (e.g., trauma, uveitis, exfoliation syndrome, pigment dispersion syndrome evident on biomicroscopic anterior segment examinations). POAG with paracentral and peripheral defects were determined manually as previously described^70^. The validity of the POAG case definition has been previously demonstrated; specifically, we showed that POAG is strongly age-related and that African ancestry and family history are risk factors^70,71^. Furthermore, our cohort has been part of large consortia used to inform the genetic architecture of POAG^2,64,72–75^.

Blood samples were collected in 1989–1990 among 32,826 NHS participants, in 1996–1999 among 29,611 NHSII participants, and in 1993–1995 among 18,159 HPFS participants. Participants arranged to have samples drawn and shipped by overnight courier to the laboratory. After sample processing, plasma aliquots were archived in liquid nitrogen freezers (≤−130 °C). POAG cases were diagnosed after blood draw until June 1, 2016 (NHS and NHSII), or January 1, 2016 (HPFS). Controls were 1:1 matched to cases on: age, cohort/gender, month and year of blood collection, time of day of blood draw, fasting status (> or ≤8 h), and race/ethnicity. Among women, we also matched on menopausal status and hormone therapy (HT) use at blood draw (premenopausal, postmenopausal and on HT, postmenopausal and not on HT, missing/unknown) and at glaucoma diagnosis. For matching factors and covariates, we used questionnaire data collected as of the blood draw, and if not available, we used biennial questionnaire data before the blood sample. The study protocol was approved by the institutional review boards (IRBs) of the Brigham and Women’s Hospital, Harvard T.H. Chan School of Public Health, and Icahn School of Medicine at Mount Sinai. Completion of self-administered questionnaires and returns of blood samples were considered as implied consent by the IRBs. Medical record release consents were obtained for the collection of medical records. This research study adhered to the tenets of the Declaration of Helsinki. Participants did not receive compensation for their involvement in this study.

### Study population – UK Biobank

The UK Biobank was approved by the National Information Governance Board for Health and Social Care and the National Health Service North West Multicenter Research Ethics Committee (reference number 06/MRE08/65). All UK Biobank participants signed electronic informed consent and did not receive compensation for their involvement in the project. This research was conducted using the UK Biobank Resource under application number 36741.

The UK Biobank is an ongoing population-based study initiated in 2006–2010 with over 500,000 participants aged 40–69 years. We used the baseline questionnaire as well as metabolomic data (http://www.ukbiobank.ac.uk). At baseline (2006–2010), participants completed a touch screen questionnaire and were considered to have glaucoma if in response to the question, “Has a doctor told you that you have any of the following problems with your eyes?”, they chose glaucoma from the menu. Participants were also considered to have glaucoma if they reported a history of glaucoma surgery or laser on the questionnaire or if they carried an ICD9/10 code for glaucoma (ICD 9: 365.*; ICD10: H40.** (excluding H40.01* and H42.*)). The UK Biobank has used this definition for glaucoma in multiple prior studies that have advanced the field, including studies where the genetic findings from another discovery dataset have been replicated in the UK Biobank;^76,77^ thus, there is strong support for the utility of the glaucoma outcome definition described above. In addition, UK Biobank studies have found similar associations with modifiable risk factors to other major glaucoma studies^78–80^.

### Metabolite profiling—NHS, NHSII, HPFS

As described previously^81–83^, plasma metabolites were profiled using liquid chromatography-tandem mass spectrometry (LC-MS) to measure endogenous, polar metabolites and lipids (Broad Institute of MIT and Harvard University (Cambridge, MA)). The metabolite profiling methods are described in detail in the supplementary materials. In total, 430 unique known metabolites were measured. We excluded 61 metabolites that were impacted by delayed blood processing^68^, leaving 369 metabolites for analyses. For metabolites with <10% missing across participant samples, missing values were imputed with 1/2 of the minimum value measured for that metabolite as has been done in prior studies^65–67^ All but one included metabolite exhibited good within-person reproducibility over 1 year, indicating that one blood sample provides a reasonable measure of longer-term exposure^68^. We assigned a metabolite class (31 in total) based on chemical taxonomy for 312 of the 369 metabolites. In total, there were 18 metabolite classes with at least 3 metabolites per class: alkaloids and derivatives (*n* = 3), steroids and steroid derivatives (*n* = 4); carnitines (*n* = 22); diglycerides (DGs, n = 14); triglycerides (TGs, *n* = 73); cholesteryl esters (*n* = 12); lysophosphatidylethanolamines (LPEs, *n* = 14); phosphatidylethanolamines (PEs, n = 13); lysophosphatidylcholines (LPCs, *n* = 17); phosphatidylcholines (PCs, *n* = 25); phosphatidylcholine plasmalogens (*n* = 13); phosphatidylethanolamines plasmalogens (*n* = 13); organoheterocyclic compounds (*n* = 12); ceramides (*n* = 4); carboxylic acids and derivatives (*n* = 14); organic acids and derivatives (*n* = 31); nucleosides, nucleotides, and analogs (*n* = 4); and sphingomyelins (*n* = 8). Metabolite values were transformed to probit scores to scale to the same range and minimize the influence of skewed distributions.

### Metabolite profiling—UK Biobank

In the UK Biobank, non-fasting baseline plasma samples from a random subset of 119,764 participants were assessed using targeted high-throughput nuclear magnetic resonance (NMR) metabolomics (Nightingale Health Ltd; Helsinki, Finland)^84^. In contrast to LC-MS, in ^1^H NMR spectroscopy, molecules with H atoms yield distinctive spectral shapes with areas under their curves proportional to the molecules’ concentration based on chemical shifts and J coupling split patterns determined from quantum mechanics^85,86^, thus allowing for detailed quantification. The platform provided quantification of 249 metabolic measures including routine clinical lipids (37 biomarkers in the panel have been certified for diagnostic use), lipoprotein subclasses, fatty acid composition, and several low-molecular-weight metabolites such as glycolysis metabolites, ketone bodies, and amino acids measured in molar concentration units. Of the 249 measures, we focused on 168 measures that were concentrations of various metabolites (the 81 remaining measures were ratios between metabolites, percentages of individual metabolites of total classes or degree of unsaturation were excluded). These 168 metabolites were mapped to 17 classes. We evaluated 14 classes which included at least 3 metabolites: amino acids (*n* = 10), triglycerides (*n* = 4), lipoprotein subclasses (*n* = 98), phospholipids (*n* = 4), lipoprotein particle concentrations (*n* = 4), total lipids (*n* = 4), cholesterol (*n* = 7), other lipids (*n* = 4), free cholesterol (*n* = 4), fatty acids (*n* = 9), cholesteryl esters (*n* = 4), lipoprotein particle sizes (*n* = 3), glycolysis related metabolites (*n* = 4), and ketone bodies (*n* = 4). Metabolite values were transformed to probit scores to scale to the same range and minimize the influence of skewed distributions.

### Model building/covariates—NHS, NHSII, HPFS

Given the matched design, we used nested multivariable-adjusted conditional logistic regression models for the analysis of individual metabolites and metabolite classes, in successive models. Model 1 did not adjust for any covariates and evaluated associations based solely on matching criteria. In Model 2, we also adjusted for factors as of blood draw that have a major influence on metabolites that included a subset of matching factors where there was imperfect matching between cases and controls (e.g., cohort/gender was perfectly matched, but age was not): age (years; matching factor), the month of blood draw (matching factor), time of blood draw (matching factor), fasting status (>8 h or less; matching factor), body mass index (BMI, kg/m^2^), smoking status (never, past, current), and physical activity (metabolic equivalents of task-hours/week). In Model 3, we further added established risk factors for POAG: glaucoma family history, ancestry (non-Hispanic White, Black, Asian, Hispanic), index of socioeconomic status based on 7 census-tract variables and for women: age at menopause (linear age). In Model 4, we added additional dietary data: intake of nitrate^87^, caffeine^88^, alcohol^71^, Alternate Healthy Eating Index excluding alcohol^89^, and total caloric intake. In Model 5, we further added co-morbidities associated with POAG: hypertension, high cholesterol, diabetes, and oral steroid use. As statins were not commercially available until after the blood draws of participants (1997), statin use was not adjusted for. We used residuals to adjust for categorical variables due to the large number of resulting cross-classified subgroups compared to the sample size when including the categorical variables in the models.

### Model building/covariates—UK Biobank

As data from the UK Biobank was used to evaluate whether the results from the NHS/NHSII/HPFS are reproduced in an independent population, we used multiple logistic regression to evaluate the association between metabolite levels in relation to prevalent glaucoma. The multiple logistic regression model adjusted for age and age-squared (years; to finely control for age), gender, ethnicity (Black, Asian, White, and other), smoking status (never, past, and current smoker), number of cigarettes smoked daily among current smokers, alcohol intake frequency (daily or almost daily, 3–4 times a week, 1–2 times a week, 1–3 times a month, and special occasions only, never, or prefer not to disclose), coffee and tea consumption (cups per day), physical activity (Metabolic Equivalent of Task (MET)-hours/week), Townsend deprivation index (range: −6 to 11; a higher index score indicates more relative poverty for a given residential area), BMI (kg/m^2^), systolic blood pressure (mm Hg), history of diabetes (yes or no), history of cardiovascular disease, systemic beta-blocker use, use of statin drugs and spherical equivalent. We included 46,961 participants (2238 glaucoma cases and 44,723 controls) with complete data on metabolites and covariates.

### Analytic approach—all cohorts

All analyses were performed with SAS 9.4 and R 4.0.3 (R packages: Biobase version 2.50, fgsea version 1.16, tableone version 0.13, ggplot2 version 3.3.5, RColorBrewer version 1.1-2, svglite 2.1.0, data.table 1.14.2, tidyverse version 1.3.1, haven version 2.4.3, labeled version 2.10.0, plyr 1.8.8, qvalue version 2.22.0, multtest version 2.46.0). For analyses of individual metabolites, metabolite values were transformed to probit scores and used as continuous variables (per 1 standard deviation (SD) increase) to calculate p-values. We estimated the odds ratios (OR) and 95% confidence intervals (CIs) per 1 SD increase in metabolite levels.

For evaluating individual metabolites, the number of effective tests (NEF)^90^ was used to adjust for multiple comparisons as NEF has the advantage of accounting for the high correlation structure of the metabolomics data. NEF < 0.05 was considered statistically significant and NEF < 0.2 was considered worthy of additional analysis given that this was an exploratory study.

For metabolite class analyses, Metabolite Set Enrichment Analysis^91^ was used. As metabolite classes overall are not correlated, to adjust for multiple comparisons, False Discovery Rate (FDR)^92^ was used. FDR < 0.05 was considered statistically significant, while FDR < 0.2 was considered nominally significant and worth considering in future analyses given the hypothesis-generating aspect of this study. All statistical tests were two-sided.

### Reporting summary

Further information on research design is available in the Nature Portfolio Reporting Summary linked to this article.

## Supplementary information

Supplementary Information

Description of Additional Supplementary Files

Supplementary Data 1-2

Reporting Summary

## Data Availability

Data from the UK Biobank cannot be shared per our Material Transfer Agreement. UK Biobank data requests can be made directly to the UK Biobank via https://www.ukbiobank.ac.uk/enable-your-research/apply-for-access. Data from the health professional cohorts (NHS, NHSII, and HPFS) are not publicly available for the following reason: data contain information that could compromise research participant privacy. Reasonable requests to access these data can be made by vision research investigators one year after publication via http://www.nurseshealthstudy.org/researchers. Investigators can expect initial responses within 4 weeks of request submission. Source data supporting all our findings (Figs. 1–5 and Supplemental Fig. 1–6) are provided with this publication as a Source Data file. Source data are provided in this paper.

## Source data

Source Data

## Code availability

The code used to conduct this study is available on GitHub at https://github.com/OanaZeleznik/POAG.
