# Supplementary material for: Plasma metabolite profile for primary open-angle glaucoma in three US cohorts and the UK Biobank
Source: Nat Commun. 2023 May 19;14:2860. doi: 10.1038/s41467-023-38466-w (PMC10199010; doi:10.1038/s41467-023-38466-w)
Supplement: Supplementary file 1 — Supplementary Information [file 41467_2023_38466_MOESM1_ESM.pdf]

# Plasma metabolite profile for primary open-angle glaucoma in three US cohorts and the UK Biobank

## SUPPLEMENTAL MATERIAL

# Table of Contents

**Supplementary methods** ........ 3

**Metabolite profiling – NHS, NHSII, HPFS** ........ 3

**Supplementary figures and tables** ........ 4

**Supplementary Figure S1. POAG association pattern among triglycerides (TGs) in NHS/NHSII/HPFS (599 cases and 599 controls) based on Model 5.** ........ 4

**Supplementary Figure S2. Secondary analysis by median age (< vs. ≥58.5 years; n=598 vs. n=600) in NHS/NHSII/HPFS.** ........ 5

**Supplementary Figure S3. Secondary analysis by gender (men vs. women; n=308 vs. 890) in NHS/NHSII/HPFS.** ... 6

**Supplementary Figure S4. Secondary analysis by BMI (< vs. ≥25 kg/m$^2$; n=649 vs. 549) in NHS/NHSII/HPFS.** ....... 7

**Supplementary Figure S5. Secondary analysis by the median time between samples collection and POAG diagnosis (DX) (<10.17 vs. ≥10.17 years; n=598 vs. 600) in NHS/NHSII/HPFS.** ........ 8

**Supplementary Figure S6. Secondary analysis by self-reported glaucoma family history (yes vs. no; n=261 vs n=882) in NHS/NHSII/HPFS.** ........ 9

**UK Biobank Eye and Vision Consortium Members** ........ 10

## Supplementary methods

### Metabolite profiling – NHS, NHSII, HPFS

Hydrophilic interaction liquid chromatography (HILIC) analyses of water soluble metabolites in the positive ionization mode were conducted using an LC-MS system comprised of a Shimadzu Nexera X2 U-HPLC (Shimadzu Corp.; Marlborough, MA) coupled to a Q Exactive mass spectrometer (Thermo Fisher Scientific; Waltham, MA). We refer to this method as HILIC-positive. Metabolites were extracted from plasma (10 µL) using 90 µL of acetonitrile/methanol/formic acid (74.9:24.9:0.2 v/v/v) containing stable isotope-labeled internal standards (valine-d8, Sigma-Aldrich; St. Louis, MO; and phenylalanine-d8, Cambridge Isotope Laboratories; Andover, MA). The samples were centrifuged (10 min, 9,000 x g, 4°C), and the supernatants were injected directly into a 150 x 2 mm, 3 µm Atlantis HILIC column (Waters; Milford, MA). The column was eluted isocratically at a flow rate of 250 µL/min with 5% mobile phase A (10 mM ammonium formate and 0.1% formic acid in water) for 0.5 minutes followed by a linear gradient to 40% mobile phase B (acetonitrile with 0.1% formic acid) over 10 minutes. MS analyses were carried out using electrospray ionization in the positive ion mode using full scan analysis over 70-800 m/z at 70,000 resolution and 3 Hz data acquisition rate. Other MS settings were: sheath gas 40, sweep gas 2, spray voltage 3.5 kV, capillary temperature 350°C, S-lens RF 40, heater temperature 300°C, micro scans 1, automatic gain control target 1e6, and maximum ion time 250 ms.

Plasma lipids were profiled using a Shimadzu Nexera X2 U-HPLC (Shimadzu Corp.; Marlborough, MA). We refer to this method as C8-positive. Lipids were extracted from plasma (10 µL) using 190 µL of isopropanol containing 1,2-didodecanoyl-sn-glycero-3-phosphocholine (Avanti Polar Lipids; Alabaster, AL). After centrifugation, supernatants were injected directly onto a 100 x 2.1 mm, 1.7 µm ACQUITY BEH C8 column (Waters; Milford, MA). The column was eluted isocratically with 80% mobile phase A (95:5:0.1 vol/vol/vol 10mM ammonium acetate/methanol/formic acid) for 1 minute followed by a linear gradient to 80% mobile-phase B (99.9:0.1 vol/vol methanol/formic acid) over 2 minutes, a linear gradient to 100% mobile phase B over 7 minutes, then 3 minutes at 100% mobile-phase B. MS analyses were carried out using electrospray ionization in the positive ion mode using full scan analysis over 200–1100 m/z at 70,000 resolution and 3 Hz data acquisition rate. Other MS settings were: sheath gas 50, in source CID 5 eV, sweep gas 5, spray voltage 3 kV, capillary temperature 300°C, S-lens RF 60, heater temperature 300°C, microscans 1, automatic gain control target 1e6, and maximum ion time 100 ms. Lipid identities were denoted by the total acyl carbon number and total double bond number.

Raw data from orbitrap mass spectrometers were processed using TraceFinder 3.3 software (Thermo Fisher Scientific; Waltham, MA) and Progenesis QI version 1.0.5165.27075 (Nonlinear Dynam-ics; Newcastle upon Tyne, UK). For analytical quality control, pooled plasma reference samples and mixtures of synthetic metabolite reference standards were analyzed at the beginning and end of sample queues to assure stable analytical performance, internal standard signals were evaluated in each sample to ensure consistent sample volume injections, and pooled plasma QC samples were inserted into the analytical queue at a frequency of 5% to evaluate analytical repeatability of each metabolite. Plasma samples were thawed on ice before aliquoting. As the aliquots for the LC-MS methods were prepared from each sample, a pooled plasma sample was created by placing an additional 10 µL aliquot from each sample into a 50 mL conical centrifuge tube. The pooled plasma sample was maintained on dry ice while samples were being aliquoted to promote rapid freezing and stored at -80 C in between sample batches until all additions were made. The pooled plasma was then thawed on ice, mixed by vortexing, and sub-aliquoted to create pooled plasma QC samples for each LC-MS method. For each method, metabolite identities were confirmed using mixtures of authentic reference standards (that were previously individually identified in human plasma based on matching retention times, m/z, and MS/MS spectra) or reference samples.

Liquid chromatography (LC), through the application of distinct stationary phase chemistries, affords reproducible separation of metabolites in complex mixtures based on their physical properties while mass spectrometry (MS) enables further resolution of metabolites on the mass-to-charge ratio and quantification over a wide linear dynamic range. These methods were developed using reference standards of each metabolite to determine chromatographic retention times and multiple reaction monitoring transitions, de-clustering potentials, and collision energies for targeted analyses. The choice of peaks and retention times depend on the behavior of the standards. These analyses are guided by the latest software supplemented by manual inspection of peaks. Dr. Clary Clish who oversees the MIT lab where this scan was performed has been doing the work of species identification for more than a decade.

## Supplementary figures and tables

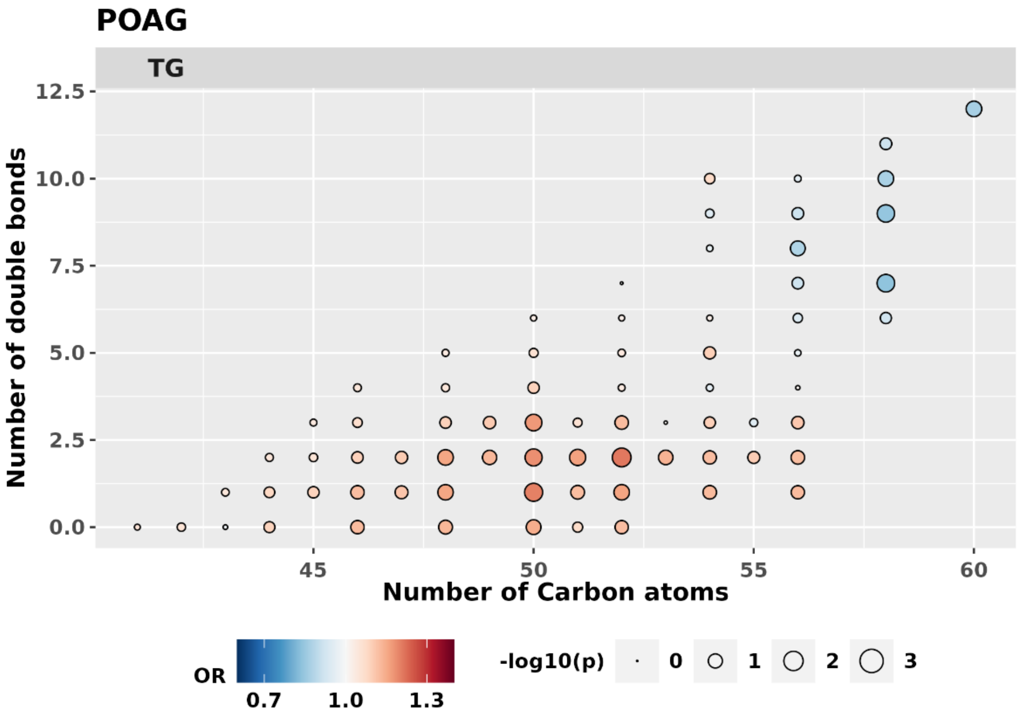


**Supplementary Figure S1. POAG association pattern among triglycerides (TGs) in NHS/NHSII/HPFS (599 cases and 599 controls) based on Model 5.**

Data are presented as odds ratios estimated with conditional logistic regression models. Model 5 included adjustment for: age + smoking status + BMI + physical activity + time of day (as matching imperfect) +month of blood draw (season, as matching imperfect) + family history of POAG + SES + race + age at menopause + nitrate intake + caffeine intake + alcohol intake + alternate healthy eating index + caloric intake + hypertension + high cholesterol + diabetes + oral/inhaled steroid use. Results are shown by the number of carbon atoms (fatty acyl chain length) and double bonds (saturation) of each triglyceride. Each triglyceride is represented by a circle. The color of the circle correlates with the association direction (red: positive, blue: inverse), and the size of the circle correlates with the statistical significance (larger circles represent smaller p-values). Source data with exact values are provided as a Source Data file.

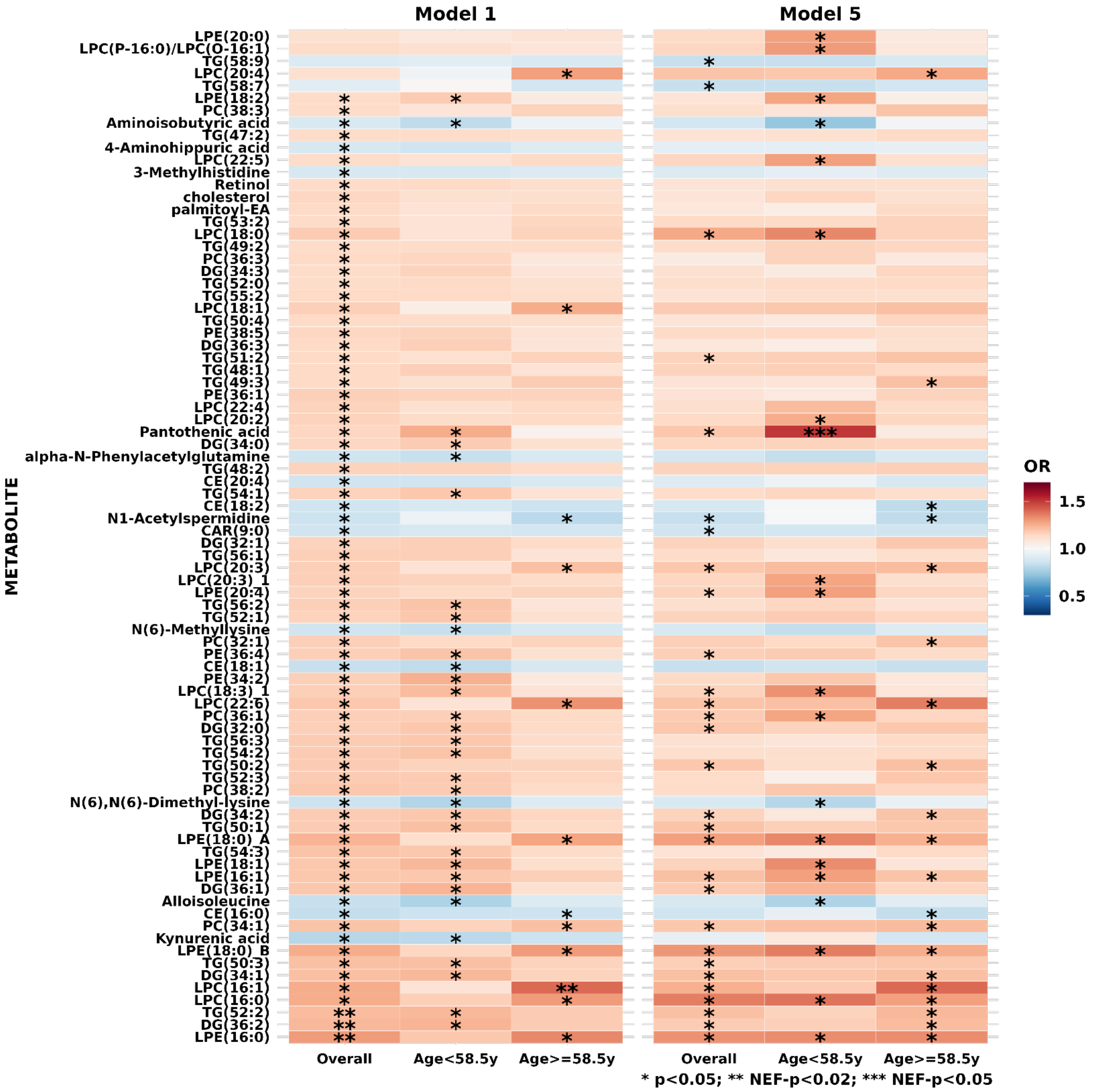


**Supplementary Figure S2. Secondary analysis by median age (< vs. ≥58.5 years; n=598 vs. n=600) in NHS/NHSII/HPFS.** 58.5 years represents the median age across all study participants. Metabolites that are nominally significant in either main Model 1 or main Model 5 are plotted. Data are presented as odds ratios estimated with logistic regression models. **Model 1**: basic model, adjusting for matching factors only; **Model 5**: age + smoking status + BMI + physical activity + time of day (as matching imperfect) +month of blood draw (season, as matching imperfect) + family history of POAG + SES + race + age at menopause + nitrate intake + caffeine intake + alcohol intake + alternate healthy eating index + caloric intake + hypertension + high cholesterol + diabetes + oral/inhaled steroid use. All statistical tests are two-sided, and we accounted for multiple comparisons by using p-values based on number of effective tests (NEF). Source data with exact values are provided as a Source Data file.

*p<0.05. ** Number of effective tests corrected (NEF)-p<0.2. *** NEF-p<0.05.

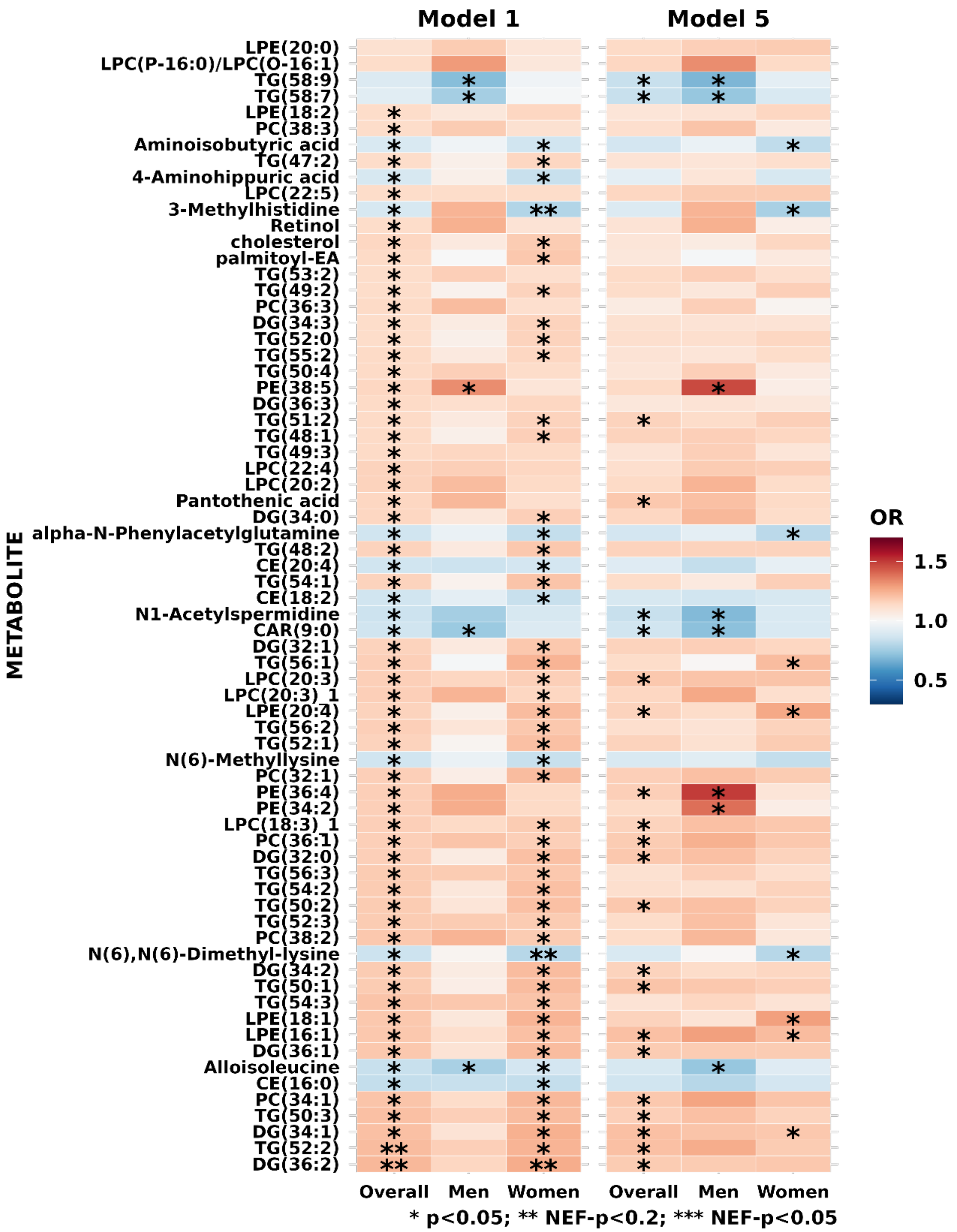


**Supplementary Figure S3. Secondary analysis by gender (men vs. women; n=308 vs. 890) in NHS/NHSII/HPFS.** Metabolites that are nominally significant in either main Model 1 or main Model 5 are plotted. Metabolites measured in women or men only are not shown. Data are presented as odds ratios estimated with conditional logistic regression models. **Model 1**: basic model, adjusting for matching factors only; **Model 5**: age + smoking status + BMI + physical activity + time of day (as matching imperfect) +month of blood draw (season, as matching imperfect) + family history of POAG + SES + race + age at menopause + nitrate intake + caffeine intake + alcohol intake + alternate healthy eating index + caloric intake + hypertension + high cholesterol + diabetes + oral/inhaled steroid use. All statistical tests are two-sided, and we accounted for multiple comparisons by using p-values based on number of effective tests (NEF). Source data with exact values are provided as a Source Data file.

*p<0.05. ** Number of effective tests corrected (NEF)-p<0.2. *** NEF-p<0.05.

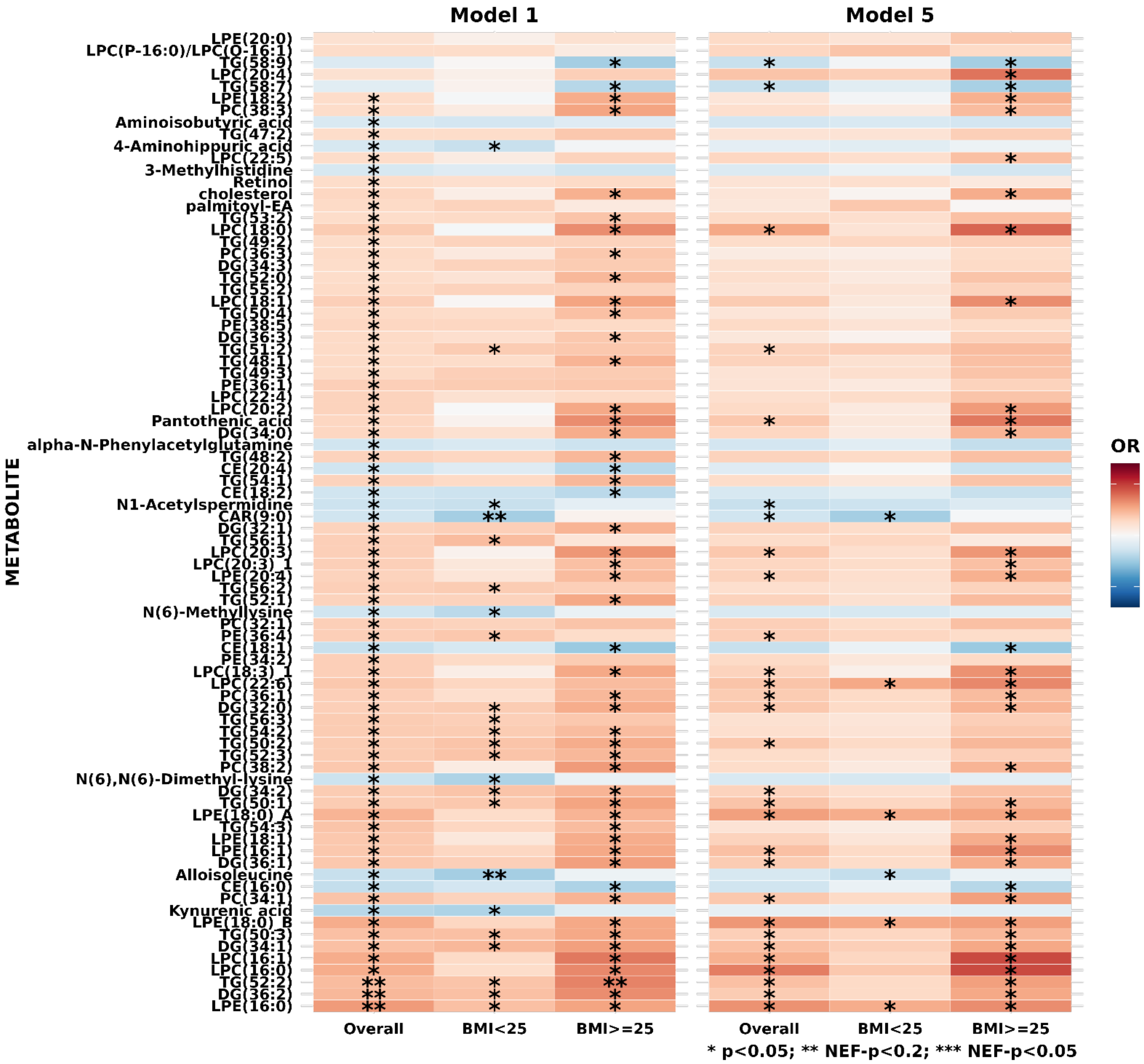


**Supplementary Figure S4. Secondary analysis by BMI (< vs. ≥25 kg/m$^2$; n=649 vs. 549) in NHS/NHSII/HPFS.** Metabolites that are nominally significant in either main Model 1 or main Model 5 are plotted. Data are presented as odds ratios estimated with logistic regression models. **Model 1**: basic model, adjusting for matching factors only; **Model 5**: age + smoking status + BMI + physical activity + time of day (as matching imperfect) +month of blood draw (season, as matching imperfect) + family history of POAG + SES + race + age at menopause + nitrate intake + caffeine intake + alcohol intake + alternate healthy eating index + caloric intake + hypertension + high cholesterol + diabetes + oral/inhaled steroid use.

All statistical tests are two-sided, and we accounted for multiple comparisons by using p-values based on number of effective tests (NEF). Source data with exact values are provided as a Source Data file.

*p<0.05. ** Number of effective tests corrected (NEF)-p<0.2. *** NEF-p<0.05.

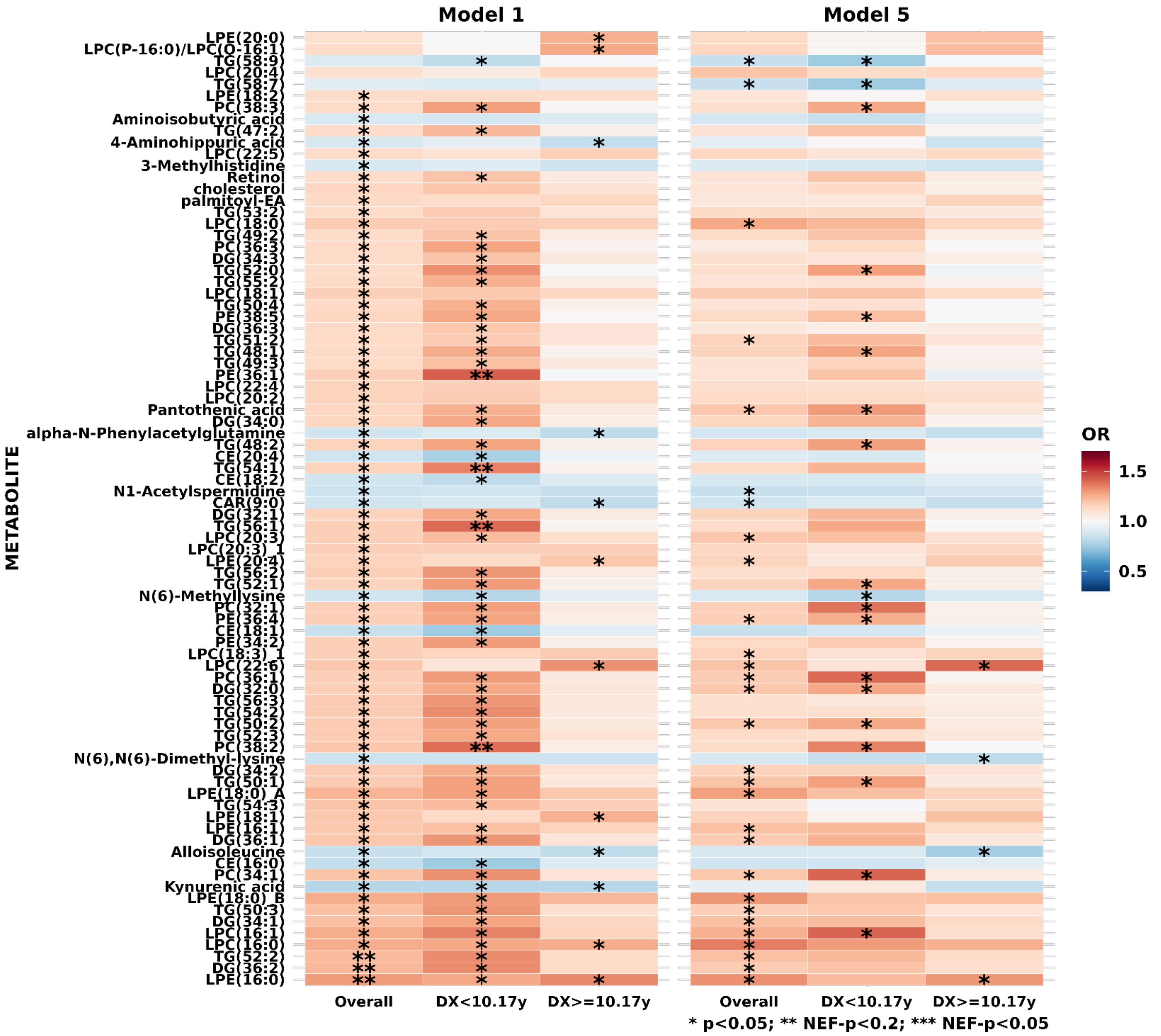


**Supplementary Figure S5. Secondary analysis by the median time between samples collection and POAG diagnosis (DX) (<10.17 vs. ≥10.17 years; n=598 vs. 600) in NHS/NHSII/HPFS.**

10.17 years represents the median time between sample collection and POAG diagnosis. Metabolites that are nominally significant in either main Model 1 or main Model 5 are plotted. Data are presented as odds ratios estimated with logistic regression models. **Model 1**: basic model, adjusting for matching factors only; **Model 5**: age + smoking status + BMI + physical activity + time of day (as matching imperfect) +month of blood draw (season, as matching imperfect) + family history of POAG + SES + race + age at menopause + nitrate intake + caffeine intake + alcohol intake + alternate healthy eating index + caloric intake + hypertension + high cholesterol + diabetes + oral/inhaled steroid use. All statistical tests are two-sided, and we accounted for multiple comparisons by using p-values based on number of effective tests (NEF). Source data with exact values are provided as a Source Data file.

*p<0.05. ** Number of effective tests corrected (NEF)-p<0.2. *** NEF-p<0.05.

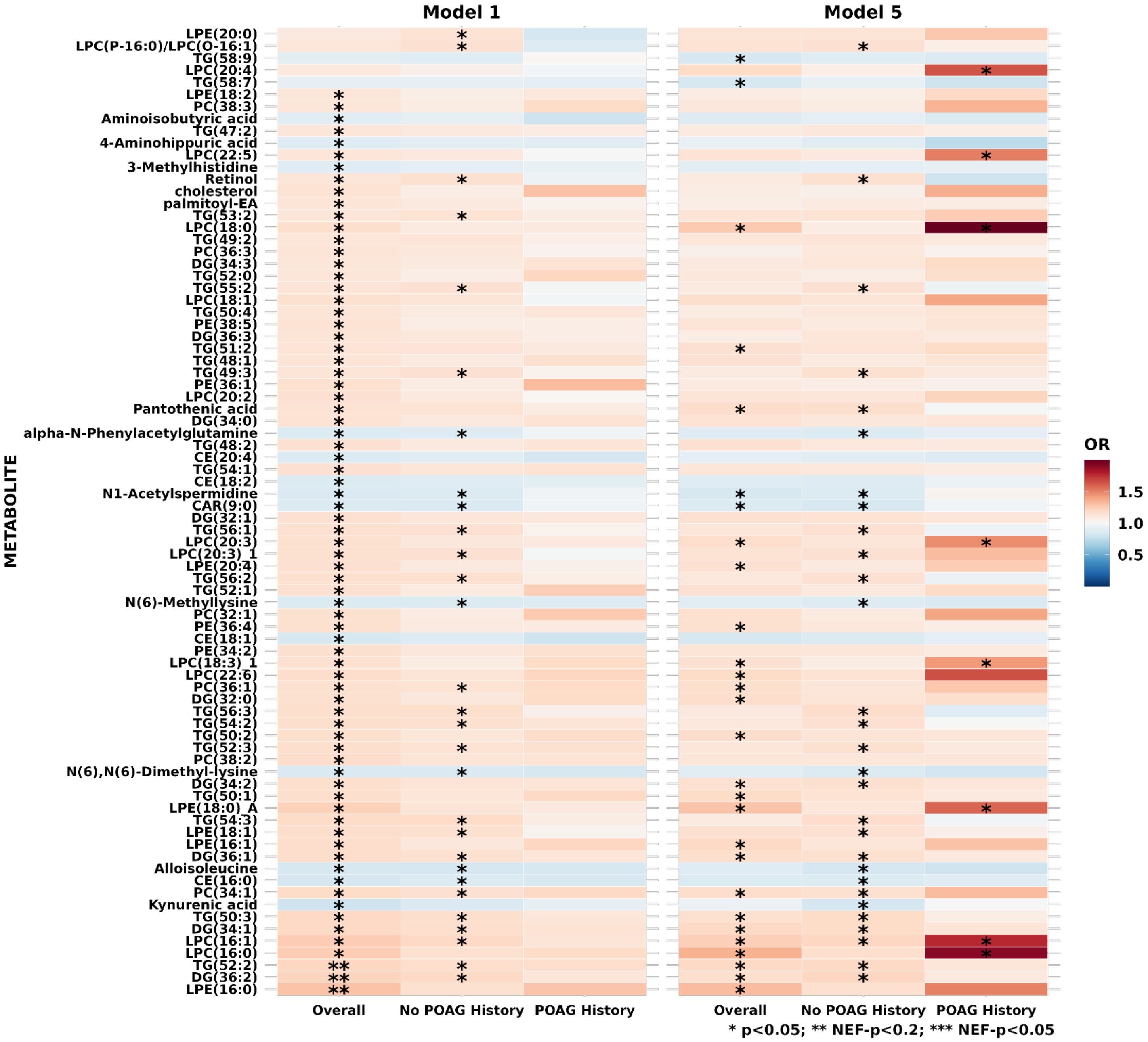


**Supplementary Figure S6. Secondary analysis by self-reported glaucoma family history (yes vs. no; n=261 vs n=882) in NHS/NHSII/HPFS.**

Metabolites that are nominally significant in either main Model 1 or main Model 5 are plotted. Data are presented as odds ratios estimated with logistic regression models. **Model 1**: basic model, adjusting for matching factors only; **Model 5**: age + smoking status + BMI + physical activity + time of day (as matching imperfect) +month of blood draw (season, as matching imperfect) + family history of POAG + SES + race + age at menopause + nitrate intake + caffeine intake + alcohol intake + alternate healthy eating index + caloric intake + hypertension + high cholesterol + diabetes + oral/inhaled steroid use.

All statistical tests are two-sided, and we accounted for multiple comparisons by using p-values based on number of effective tests (NEF). Source data with exact values are provided as a Source Data file.

*p<0.05. ** Number of effective tests corrected (NEF)-p<0.2. *** NEF-p<0.05.

**UK Biobank Eye and Vision Consortium Members**


Naomi Allen, Nuffield Department of Population Health, University of Oxford, Oxford, UK.
Tariq Aslam, Manchester Royal Eye Hospital, The University of Manchester, Manchester, UK.
Denize Atan, Bristol Eye Hospital, University of Bristol, Bristol, UK.
Konstantinos Balaskas, Moorfields Eye Hospital, London, UK.
Sarah Barman, Department of Computer Science and Mathematics, Kingston University, London, UK.
Jenny Barrett, School of Medicine, University of Leeds, Leeds, UK.
Paul Bishop, Division of Evolution, Infection and Genomics, The University of Manchester, Manchester, UK.
Graeme Black, Division of Evolution, Infection and Genomics, The University of Manchester, Manchester, UK.
Tasanee Braithwaite, Guy’s and St Thomas' Hospital NHS Foundation Trust, London, UK.
Roxana Carare, University of Southampton, Southampton, UK.
Usha Chakravarthy, Ophthalmology and Vision Sciences, Royal Victoria Hospital (The Belfast Trust) and Queens University of Belfast, Belfast, UK.
Michelle Chan, Moorfields Eye Hospital, London, UK.
Sharon Chua, University College London Institute of Ophthalmology, London, UK.
Alexander Day, Moorfields Eye Hospital, London, UK.
Parul Desai, Moorfields Eye Hospital, London, UK.
Baljean Dhillon, Centre for Clinical Brain Sciences, University of Edinburgh, Edinburgh, Scotland.
Andrew Dick, Bristol Eye Hospital, University Hospitals Bristol and Weston, Bristol, UK.
Alexander Doney, Pat Macpherson Centre for Pharmacogenomics and Pharmacogenetics, Division of Population Health & Genomics, School of Medicine, University of Dundee, Dundee, UK.
Cathy Egan, Moorfields Eye Hospital, London, UK.
Sarah Ennis, Human Development and Health, Faculty of Medicine, University Hospital Southampton, Southampton, Hampshire, UK.
Paul Foster, NIHR Biomedical Research Centre at Moorfields Eye Hospital and UCL Institute of Ophthalmology, London, UK.
Marcus Fruttiger, University College London Institute of Ophthalmology, London, UK.
John Gallacher, Dementias Platform UK, Oxford, UK.
David (Ted) Garway-Heath, National Institute for Health Research Biomedical Research Centre, Moorfields Eye Hospital NHS Foundation Trust and University College London Institute of Ophthalmology, London, UK.
Jane Gibson, Southampton General Hospital, University of Southampton, Southampton, UK.
Jeremy Guggenheim, School of Optometry & Vision Sciences, Cardiff University, Cardiff, UK.
Chris Hammond, Department of Twin Research and Genetic Epidemiology, King's College London, London, UK.
Alison Hardcastle, University College London Institute of Ophthalmology, London, UK.
Simon Harding, Department of Eye and Vision Science, Institute of Life Course and Medical Sciences, University of Liverpool, and St. Paul's Eye Unit. Liverpool University Hospitals, Liverpool, UK.
Ruth Hogg, Centre for Public Health, School of Medicine, Dentistry and Biomedical Sciences, Belfast, UK.
Pirro Hysi, Department of Twin Research and Genetic Epidemiology, King's College London, London, UK.
Pearse Keane, National Institute for Health Research Biomedical Research Centre, Moorfields Eye Hospital NHS Foundation Trust & UCL, Institute of Ophthalmology, London, UK.
Peng Tee Khaw, National Institute for Health Research Biomedical Research Centre at Moorfields Eye Hospital, UCL Institute of Ophthalmology, London, UK.
Anthony Khawaja, National Institute for Health Research Biomedical Research Centre at Moorfields Eye Hospital, UCL Institute of Ophthalmology, London, UK.
Gerassimos Lascaratos, Department of Ophthalmology, King's College Hospital NHS Foundation Trust, London, UK.
Thomas Littlejohns, Nuffield Department of Population Health, University of Oxford, Oxford, UK.
Andrew Lotery, Clinical and Experimental Sciences, Faculty of Medicine, University of Southampton, Southampton, UK.
Robert Luben, National Institute for Health Research Biomedical Research Centre, Moorfields Eye Hospital NHS Foundation Trust and UCL Institute of Ophthalmology, London, UK.
Phil Luthert, University College London Institute of Ophthalmology and National Institute for Health Research Biomedical Research Centre for Ophthalmology at Moorfields Eye Hospital and University College London Institute of Ophthalmology, London, UK.
Tom MacGillivray, Centre for Clinical Brain Sciences, University of Edinburgh, Edinburgh, UK.
Sarah Mackie, Leeds Biomedical Research Centre, Leeds Teaching Hospitals NHS Trust, Leeds, UK.
Savita Madhusudhan, Royal Liverpool and Broadgreen University Hospitals NHS Trust, Liverpool, UK.
Bernadette McGuinness, Centre for Public Health, School of Medicine, Dentistry and Biomedical Sciences, Queen's University Belfast,

Belfast, Northern Ireland, UK.
Gareth McKay, Centre for Public Health, School of Medicine, Dentistry and Biomedical Sciences, Queen's University, Belfast, UK.
Martin McKibbin, Leeds Teaching Hospitals NHS Trust, Leeds, UK.
Tony Moore, University College London Institute of Ophthalmology and NIHR Biomedical Research Centre for Ophthalmology at Moorfields Eye Hospital and University College London Institute of Ophthalmology, London, UK.
James Morgan, School of Optometry & Vision Sciences, Cardiff University, Cardiff, UK.
Eoin O'Sullivan, King's College Hospital, London, UK.
Richard Oram, University of Exeter College of Medicine & Health, Exeter, UK.
Chris Owen, Population Health Research Institute St George's, University of London, London, UK.
Praveen Patel, National Institute for Health Research Biomedical Research Centre at Moorfields Eye Hospital, University College London Institute of Ophthalmology, London, UK.
Euan Paterson, Queen's University Belfast, Belfast, Northern Ireland, UK.
Tunde Peto, Centre for Public Health, Queen's University Belfast, Belfast, UK.
Axel Petzold, The National Hospital for Neurology and Neurosurgery & Moorfields Eye Hospital, London, UK.
Nikolas Pontikos, National Institute for Health Research Biomedical Research Centre, Moorfields Eye Hospital NHS Foundation Trust & UCL, Institute of Ophthalmology, London, UK.
Jugnoo Rahi, Population, Policy and Practice Research and Teaching Department, Great Ormond Street Institute of Child Health, University College London, London, UK.
Alicja Rudnicka, Population Health Research Institute, St George's, University of London, London, UK.
Naveed Sattar, School of Cardiovascular and Metabolic Health, University of Glasgow, Glasgow, UK.
Jay Self, Clinical and Experimental Sciences, School of Medicine, University of Southampton, Southampton, UK.
Panagiotis Sergouniotis, The University of Manchester, Manchester, UK.
Sobha Sivaprasad, Moorfields Eye Unit, National Institute for Health Research, London, UK.
David Steel, Biosciences Institute, Faculty of Medical Sciences, Newcastle University, Newcastle upon Tyne, UK
Irene Stratton, Gloucestershire Retinal Research Group, Cheltenham General Hospital, Gloucestershire Hospitals NHS Foundation Trust, Cheltenham, UK.
Nicholas Strouthidis, National Institute of Health Research, Moorfields Eye Hospital, London, UK.
Cathie Sudlow, Centre for Medical Informatics, Usher Institute of Population Health Sciences and Informatics, University of Edinburgh, UK.
Zihan Sun, University College London Institute of Ophthalmology, London, UK.
Robyn Tapp, Research Centre for Intelligent Health Care, Coventry University, Coventry, UK.
Dhanes Thomas, NIHR Biomedical Research Centre at Moorfields Eye Hospital, UCL Institute of Ophthalmology, London, UK.
Emanuele Trucco, School of Science and Engineering, University of Dundee, Dundee, UK.
Adnan Tufail, Moorfields Eye Hospital NHS Foundation Trust, London, UK.
Ananth Viswanathan, Moorfields Eye Hospital NHS Foundation Trust, London, UK.
Veronique Vitart, MRC Human Genetics Unit, Institute of Genetics and Cancer, University of Edinburgh, Western General Hospital, Edinburgh, UK.
Mike Weedon, Department of Clinical and Biomedical Sciences, Faculty of Health and Life Sciences, University of Exeter, Exeter, UK.
Katie Williams, King's College London, London, UK.
Cathy Williams, Bristol Eye Hospital, University of Bristol, Bristol, UK.
Jayne Woodside, Centre for Public Health, School of Medicine, Dentistry and Biomedical Sciences, Institute of Clinical Science, Queen's University Belfast, Belfast, UK.
Max Yates, Norwich Epidemiology Centre, Norwich Medical School, University of East Anglia, Norwich, UK.
Yalin Zheng, Institute of Life Course and Medical Science, University of Liverpool, Liverpool UK.
