# Supplementary material for: Plasma metabolite profile for primary open-angle glaucoma in three US cohorts and the UK Biobank
Source: Nat Commun. 2023 May 19;14:2860. doi: 10.1038/s41467-023-38466-w (PMC10199010; doi:10.1038/s41467-023-38466-w)
Supplement: Supplementary file 2 — Description of Additional Supplementary Files [file 41467_2023_38466_MOESM2_ESM.pdf]

Description of Additional Supplementary Files

Supplementary Data 1: Odds ratios (OR) and 95% confidence intervals (CI) of POAG for all metabolites in Model 1 and Model 5 in NHS, NHS2, HPFS (599 POAG cases and 599 controls

Supplementary Data 2: Odds ratios (OR) and 95% confidence intervals (CI) of glaucoma for all metabolites in multivariable-adjusted model in the UK Biobank (2238 glaucoma cases, 44723 controls)
