# Supplementary material for: Plasma metabolite profile for primary open-angle glaucoma in three US cohorts and the UK Biobank
Source: Nat Commun. 2023 May 19;14:2860. doi: 10.1038/s41467-023-38466-w (PMC10199010; doi:10.1038/s41467-023-38466-w)
Supplement: Supplementary file 4 — Reporting Summary [file 41467_2023_38466_MOESM4_ESM.pdf]

nature portfolio

Corresponding author(s): Oana Zeleznik

Last updated by author(s): Mar 16, 2023

Reporting Summary

Nature Portfolio wishes to improve the reproducibility of the work that we publish. This form provides structure for consistency and transparency in reporting. For further information on Nature Portfolio policies, see our Editorial Policies and the Editorial Policy Checklist.

Statistics

For all statistical analyses, confirm that the following items are present in the figure legend, table legend, main text, or Methods section.

n/a	Confirmed	
☐	☒	The exact sample size (*n*) for each experimental group/condition, given as a discrete number and unit of measurement
☐	☒	A statement on whether measurements were taken from distinct samples or whether the same sample was measured repeatedly
☐	☒	The statistical test(s) used AND whether they are one- or two-sided<br>*Only common tests should be described solely by name; describe more complex techniques in the Methods section.*
☐	☒	A description of all covariates tested
☐	☒	A description of any assumptions or corrections, such as tests of normality and adjustment for multiple comparisons
☐	☒	A full description of the statistical parameters including central tendency (e.g. means) or other basic estimates (e.g. regression coefficient) AND variation (e.g. standard deviation) or associated estimates of uncertainty (e.g. confidence intervals)
☐	☒	For null hypothesis testing, the test statistic (e.g. *F*, *t*, *r*) with confidence intervals, effect sizes, degrees of freedom and *P* value noted<br>*Give P values as exact values whenever suitable.*
☒	☐	For Bayesian analysis, information on the choice of priors and Markov chain Monte Carlo settings
☒	☐	For hierarchical and complex designs, identification of the appropriate level for tests and full reporting of outcomes
☒	☐	Estimates of effect sizes (e.g. Cohen's *d*, Pearson's *r*), indicating how they were calculated

Our web collection on statistics for biologists contains articles on many of the points above.

Software and code

Policy information about availability of computer code

Data collection	TraceFinder versions 3.3 (Thermo Fisher, Waltham MA) and Progenesis QI version 1.0.5165.27075 (Nonlinear Dynamics, Durham NC). For UK Biobank data, please refer to: https://www.ukbiobank.ac.uk/enable-your-research
Data analysis	R version 4.0.3, SAS 9.4;<br>R packages: Biobase version 2.50, fgsea version 1.16, tableone version 0.13, ggplot2 version 3.3.5, RColorBrewer version 1.1-2, svglite 2.1.0, data.table 1.14.2, tidyverse version 1.3.1, haven version 2.4.3, labelled version 2.10.0, plyr 1.8.8, qvalue version 2.22.0, multtest version 2.46.0<br>Code: https://github.com/OanaZeleznik/POAG

For manuscripts utilizing custom algorithms or software that are central to the research but not yet described in published literature, software must be made available to editors and reviewers. We strongly encourage code deposition in a community repository (e.g. GitHub). See the Nature Portfolio guidelines for submitting code & software for further information.

Data

Policy information about availability of data

All manuscripts must include a data availability statement. This statement should provide the following information, where applicable:

- Accession codes, unique identifiers, or web links for publicly available datasets
- A description of any restrictions on data availability
- For clinical datasets or third party data, please ensure that the statement adheres to our policy

Raw data from our health professional cohorts are available upon reasonable request to the corresponding author via email by vision research investigators one year after publication. Source data supporting our findings (Figures 1-5 and Supplemental Figures 1-6) are provided with this publication as a Source Data file.
Raw data from the UK Biobank cannot be shared per our Material Transfer Agreement

Human research participants

Policy information about studies involving human research participants and Sex and Gender in Research.

Reporting on sex and gender

This study includes analyses stratified by self-reported biological sex. We do not have information on gender. We conducted analyses among women by combining data from NHS and NHSII and among men based on data from HPFS (Supplementary Figure S3). As we did not identify any statistically significant difference by sex in the main analysis, we did not conduct a stratified analysis by sex in the replication dataset, the UK Biobank.

Population characteristics

In NHS, NHSII and HPFS, among 599 cases, 74.3% were female, with a mean (SD) age at blood draw of 58.0 (SD=8.0) years and at diagnosis of 68.3 (SD=9.2) years. Mean time between blood draw to diagnosis was 10.3 years. Controls were similar to cases for the matching factors. In the UK Biobank, the mean age was 58 (SD=8), with 54% women and 92% white. Distributions of POAG risk factors were generally in the expected directions for cases and controls.

Recruitment

Our samples are part of prospective matched case-control studies nested within the Nurses Health Studies' (NHS), NHSII and Health Professional Follow-up Study (HPFS). NHS, NHSII and HPFS are closed cohorts. NHS and NHSII include registered female nurses (RN) while HPFS includes male health professionals (veterinarians, dentists, pharmacists, optometrists, osteopath physicians, and podiatrists). Although the participants had a slightly higher socioeconomic status than did the general population and were mostly White, which may have initially affected generalizability, the population selection enhances internal validity because the health knowledge and commitment to research of the health professionals contributes to high-quality and complete self-reported health data as well as high follow-up rates. Although our participants are not a random U.S. sample, it seems unlikely that the biological relations in these participants will differ from the general population. The nurses and health professionals represent all but the poorest strata of society, and except for being predominantly Caucasian (reflecting registered nurses and health professionals at enrollment), have similar age and region-adjusted chronic disease rates to national rates, suggesting that our study is reasonably representative. Additionally, we conducted a replication analysis in an independent UK population.

Ethics oversight

The study protocol was approved by the institutional review boards (IRBs) of the Brigham and Women's Hospital, Harvard T.H. Chan School of Public Health, and Icahn School of Medicine at Mount Sinai. Completion of self-administered questionnaires and returns of blood samples were considered as implied consents by the IRBs. Medical record release consents were obtained for collection of medical records. This research study adhered to the tenets of the Declaration of Helsinki. Participants did not receive compensation for their involvement in this study.

The UK Biobank was approved by the National Information Governance Board for Health and Social Care and the National Health Service North West Multicenter Research Ethics Committee (reference number 06/MRE08/65). All UK Biobank participants signed electronic informed consent and did not receive compensation for their involvement in the project. This research was conducted using the UK Biobank Resource under application number 36741.

Note that full information on the approval of the study protocol must also be provided in the manuscript.

Field-specific reporting

Please select the one below that is the best fit for your research. If you are not sure, read the appropriate sections before making your selection.

- [x] Life sciences
- [ ] Behavioural & social sciences
- [ ] Ecological, evolutionary & environmental sciences

For a reference copy of the document with all sections, see nature.com/documents/nr-reporting-summary-flat.pdf

Life sciences study design

All studies must disclose on these points even when the disclosure is negative.

Sample size

NHS, NHSII and HPFS sample size was limited by the study budget and the number of cases diagnosed after an eligible blood draw through the time of assay who developed confirmed POAG through June 2016 (NHS and NHSII) and January 2016 (HPFS). Based on this, 599 cases and 599

controls were sent for assay. In the research proposal, a power calculation was conducted based on 500 expected cases and 500 controls, based on observed cases plus cases using cohort and age-specific incidence rates, accounting for aging, from after blood draw through 2016. With 300 metabolites, we expected to have >80% power to detect an effect size as low as 0.3, which is the case versus control difference in a given metabolite of as small as 0.3 SDs for a non-targeted approach, even if we use a conservative Bonferroni correction (=0.05/ 300)

UK Biobank sample size was limited to samples with available data on outcome and measured metabolomic profiles. In the research proposal, a power calculation was conducted based on 2000 expected cases, and 40000 expected controls, which would provide >80% power to detect an effect size as low as 0.064 SDs.

Data exclusions	We excluded samples which did not have data on the outcome or metabolomic profiles as this makes the analysis impossible.
Replication	Results for NHS, NHSII and HPFS were replicated in an independent dataset, the UKBiobank.
Randomization	Samples from the NHS, NHSII and HPFS were run together, with matched case-control pairs (as sets) distributed randomly within the batch, and the order of the case and controls within each pair randomly assigned. Therefore, the case and its control were always directly adjacent to each other in the analytic run, thereby limiting variability in platform performance across matched case-control pairs. In addition, >10% quality control (QC) samples, to which the laboratory was blinded, were also profiled. These were randomly distributed among the participants' samples. From the entire UK Biobank population, a random subset of non-fasting baseline plasma samples (aliquot 3) from 118, 466 individuals and 1298 repeat-visit samples were measured using high-throughput NMR spectroscopy (Nightingale Health Plc; biomarker quantification version 2020).
Blinding	The laboratory in which NHS, NHSII and HPFS metabolomics data was measured and preprocessed was blinded to QC/participant status and to the case/control status of the samples.<br><br>In the UK Biobank, metabolite measurements were conducted blinded prior to the linkage to the UK Biobank health outcomes. The metabolic biomarker data were curated and linked to UK Biobank clinical data in late-May 2020.

Reporting for specific materials, systems and methods

We require information from authors about some types of materials, experimental systems and methods used in many studies. Here, indicate whether each material, system or method listed is relevant to your study. If you are not sure if a list item applies to your research, read the appropriate section before selecting a response.

Materials & experimental systems

n/a	Involved in the study	
☒	☐	Antibodies
☒	☐	Eukaryotic cell lines
☒	☐	Palaeontology and archaeology
☒	☐	Animals and other organisms
☒	☐	Clinical data
☒	☐	Dual use research of concern

Methods

n/a	Involved in the study	
☒	☐	ChIP-seq
☒	☐	Flow cytometry
☒	☐	MRI-based neuroimaging
